# Achieving the robust immobilization of CoP nanoparticles in cellulose nanofiber network-derived carbon *via* chemical bonding for a stable potassium ion storage†

**DOI:** 10.1039/d0ra09478a

**Published:** 2020-12-17

**Authors:** Xudong Zhao, Dan Zhou, Mingyang Chen, Jiaqi Yang, Li-Zhen Fan

**Affiliations:** Center for Green Innovation, School of Mathematics and Physics, Beijing Advanced Innovation Center for Materials Genome Engineering, University of Science and Technology Beijing Beijing 100083 China zhoudan@ustb.edu.cn fanlizhen@ustb.edu.cn; Center for Green Innovation, School of Materials Science and Engineering, University of Science and Technology Beijing Beijing 100083 China mychen@ustb.edu.cn; Office of Educational Administration, Shenyang Open University Shenyang 110003 China; Shunde Graduate School of University of Science and Technology Beijing Foshan 528000 China; Beijing Computational Science Research Center Beijing 100084 China

## Abstract

Potassium-ion batteries (KIBs) are currently being investigated as a potential alternative to lithium-ion batteries (LIBs) because of the natural abundance of K resources. Presently, it is crucial yet challenging to explore suitable anode materials for stable K-storage. Herein, a novel robust CoP–carbon composite with highly dispersed CoP nanoparticles (NPs) immobilized in natural cellulose nanofiber network (CNF)-derived carbon (denoted as CoP@CNFC) is synthesized *via* chemical bonding through a facile hydrothermal and subsequent *in situ* phosphidation approach. The designed structure can provide diverse merits, including fast reaction kinetics, sufficient active sites and effective accommodation for K^+^ insertion/extraction; thus, CoP@CNFC delivers desired electrochemical performance, including considerable reversible capacity, enhanced rate capability and excellent cycling stability. Additionally, the electrochemical reaction mechanism of CoP@CNFC was clearly revealed by *ex situ* characterizations and theoretical simulations of cyclic voltammetry (CV) and solid electrolyte interface (SEI) profiles based on first-principles calculations. The achieved deep elucidation of the reversible process of K^+^ insertion and extraction on the surface/interface of the active material during the discharge and charge states clearly highlights its significance for stable K-storage. This work promotes the facile design and deep understanding of nanostructured high-capacity electrodes of transition metal phosphates for rechargeable KIBs.

## Abstract 2

A robust CoP@CNFC with enhanced electrochemical performance was synthesized *via* chemical bonding and demonstrates a promising potential toward an efficient K-storage.

## Graphical abstract

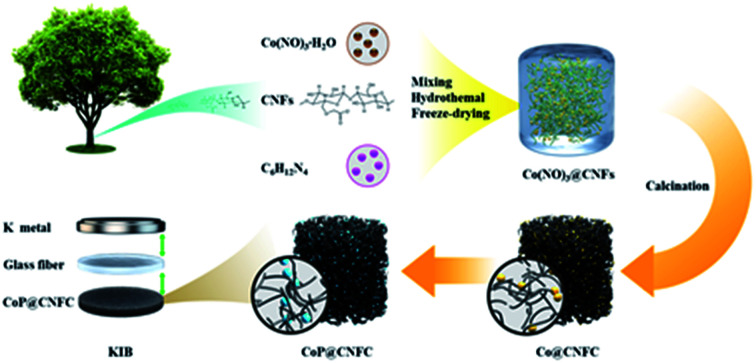

## 1. Introduction

With the persistently increasing demand for lithium-ion batteries (LIBs) for portable electronics and electric vehicles, the accelerated consumption and high cost of lithium resources has become a severe issue to address.^1–3^ Recently, tremendous efforts have been devoted to exploring new types of rechargeable batteries beyond LIBs on the basis of low cost and abundant resources. To this end, potassium-ion batteries (KIBs) have received extensive attention because of the natural abundance of K resource and their identical operating principle to LIBs. Additionally, the redox potential of K^+^/K (−2.93 V *vs.* SHE) is close to that of Li^+^/Li (−3.04 V *vs.* SHE); thus, KIBs have a promising potential in the achievement of high operating voltage and energy density.^4–7^ However, the development of advanced anode materials for KIBs is still a critical challenge due to the larger ionic radius of K^+^ (1.38 Å) compared to Li^+^ (0.76 Å), which can cause substantial structural distortions and a sluggish diffusion rate during the potassiation/depotassiation process, leading to low reversible capacity, poor cycling stability and limited rate capability.^8,9^

Transition-metal phosphides (TMPs), a significant family of functional materials, are presently recognized as potential anode materials for KIBs due to their abundant raw materials and high theoretical capacity based on the conversion reaction mechanism.^10–13^ Moreover, TMPs demonstrate advantageous operating potential and electronic conductivity compared to their oxide counterparts.^14^ However, the practical application of TMPs is still hindered by the large volume variation-induced structural degradation upon the K^+^ insertion/extraction process, which may result in severe capacity decay and weak cycling stability.^15^ In general, two feasible strategies, namely designing nanostructured materials (*e.g.* nanoparticles, nanocubes, hollow spheres, nanowires) and integrating them with conductive carbon components (carbon nanotubes, carbon fibers and porous carbon networks) are mainly employed to improve the K-storage performance of TMPs; these strategies can offer substantial combined merits, including enhanced reaction kinetics and strengthened tolerance of mechanical strain.^3,10–16^

Notably, constructing novel robust and high-capacity electrode materials with strong interface joins *via* chemical bonding has attracted significant attention. As reported, the desired electrostatic interaction of positively charged transition metal ions and negatively charged oxidation functional groups on the carbonaceous surface can lead to the formation of chemical bonds between the transition metal and carbon,^17–20^ forming a robust transition metal–carbon composite. The composite can ensure a close connection between the transition metal and carbon materials, offer a short and effective diffusion pathway for enhanced electrochemical kinetics, and sustain the structural integrity of the electrode *via* buffering the volume variation, promoting the electrochemical performance. Inspired by this, by treating the metal–carbon composite with subsequent phosphating, a robust TMP–carbon composite with TMP nanoparticles (NPs) highly dispersed in carbon materials can be obtained. This unique design tactic is significant and feasible for efficient K-storage. However, owing to the lack of suitable preparation methods and precursor materials, novel TMP–carbon composites have rarely been reported.

Herein, we have developed a facile hydrothermal and subsequent *in situ* phosphidation route to fabricate anode materials with highly dispersed CoP NPs immobilized in natural cellulose nanofiber network (CNF)-derived carbon (denoted as CoP@CNFC). Cobalt nitrate, which can provide abundant cobalt ions in aqueous solution, was used as the positively charged TMP precursor. CNFs, which were obtained from TEMPO-induced renewable biomass and possessed numerous carboxyl and hydroxyl groups on their surfaces,^21,22^ were employed as the negatively charged carbon precursor. During the first step, strong chemical bonding induced by electrostatic interaction of the oppositely charged species was facilely generated, leading to a hybridized sol–gel composed of cobalt ions uniformly absorbed onto the surface of the CNFs. After treating the sol–gel with freeze-drying and annealing, the CNFs were converted to 3D network-like carbon (CNFC), and highly dispersed Co NPs were accordingly obtained and immobilized in the carbon matrix *via in situ* carbothermal reduction of the decomposed cobalt nitrate at high temperature, achieving the Co@CNFC composite. In the subsequent step, the Co NPs in Co@CNFC were subjected to a phosphidation process, leading to the formation of CoP@CNFC. Benefiting from favorable merits, including large specific surface areas, high conductivity, and robust structure for K-storage, the CoP@CNFC anode could deliver excellent K-storage performance, including high specific capacity, enhanced rate capability and robust cycling stability. Furthermore, *ex situ* characterizations and theoretical simulations of cyclic voltammetry (CV) and solid electrolyte interface (SEI) profiles based on first-principles calculations were combined to fundamentally understand the electrochemical reaction mechanism of the electrode material.

## 2. Experimental section

### 2.1 Synthesis of the Co@CNFC composite

Typically, 0.582 g of Co(NO_3_)_2_·6H_2_O and 1.12 g of hexamethylenetetramine (C_6_H_12_N_4_) were firstly dissolved in 40 g of CNFs aqueous solution (0.5% concentration) under ultrasound with auxiliary stirring for at least 2 h. Then, the formed homogeneous solution was transferred into a 50 mL Teflon-lined autoclave and heated at 120 °C for 6 h. After cooling to room temperature, a sol–gel solution was collected after centrifugation, followed by washing with deionized water several times. Afterwards, the sol–gel solution was further freeze-dried under vacuum for several hours to form a Co@CNFC aerogel precursor. Finally, the Co@CNFC aerogel precursor was annealed at 800 °C for 2 h with a ramping rate of 5 °C min^−1^ under air atmosphere, and the Co@CNFC composite was obtained.

### 2.2 Synthesis of CoP@CNFC composite

CoP@CNFC composite was obtained by a classical phosphidation route. Specifically, the as-prepared Co@CNFC and NaH_2_PO_2_·H_2_O (1 : 10 in m/m) were separately placed in an open quartz boat and heated in a tube furnace at 300 °C for 2 h (2 °C min^−1^) under argon atmosphere. The resulting product is the so-called CoP@CNFC composite. As a reference, CoP NPs were prepared by direct phosphidation of Co NPs under an identical annealing procedure. CNFC was obtained by the same process as CoP@CNFC without the addition of Co(NO_3_)_2_·6H_2_O, C_6_H_12_N_4_ and NaH_2_PO_2_·H_2_O.

### 2.3 Material characterizations

The X-ray diffraction (XRD) patterns of the as-synthesized samples were acquired using an X-ray diffractometer (Rigaku D/max-Rb) with Cu Kα radiation (*λ* = 0.15418 nm). The morphologies and microstructures were detected by field emission scanning electron microscopy (SEM, JEOL JSM-6330) and transmission electron microscopy (TEM, JEOL JEM-2011) equipped with energy disperse spectroscopy (EDS). The nitrogen adsorption–desorption isotherms were determined to investigate the Brunauer–Emmett–Teller (BET) specific surface area and Barrett–Joyner–Halenda (BJH) pore distribution by a gas adsorption instrument (MicroActive ASAP 2460) at 77 K. The surface chemical states were determined by X-ray photoelectron spectroscopy (ESCALAB 250Xi). The weight percentage of each composition was measured by a thermogravimetric analyzer (PerkinElmer 2400II) from the ambient temperature to 900 °C with a heating rate of 10 C min^−1^ under air atmosphere. The Raman spectra were analyzed by a RAM HR800 (532 nm).

### 2.4 Electrochemical measurements

The electrochemical properties of the as-synthesized samples were measured in CR 2032 coin cells at room temperature. A slurry containing 80 wt% of active materials, 10 wt% of Super P carbon, and 10 wt% of polyvinylidene fluoride binder was cast onto a Cu current collector, followed by drying at 100 °C for 12 h in a vacuum oven. By virtue of a subsequent punching process, a working electrode of a 12 mm disc with a mass loading of about 0.8–1.0 mg cm^−2^ was obtained. Potassium foil was used as the counter electrode, and glass fiber (GF/A, Whatman) was used as the separator. The electrolyte was obtained by dissolving 0.8 M KPF_6_ in a mixture of ethylene carbonate (EC)/diethyl carbonate (DEC) (1 : 1 vol/vol). All cells were assembled in an argon gas-filled glove box (H_2_O and O_2_ levels <0.1 ppm). A CHI760E electrochemical workstation (Chen Hua Company) was used to measure the cyclic voltammetry (CV) curves (scan rate: 0.02 mV^−1^) and electrochemical impedance spectroscopy (EIS) (amplitude: 5 mV, frequency: 100 kHz to 0.01 Hz) in the voltage range of 0.01 to 3.0 V. Galvanostatic charge/discharge (GCD) tests were collected using a LAND CT2001A battery testing instrument between 0.01 and 3.0 V with various current densities.

### 2.5 Computational details

The electrochemical reactions of the CoP anode material in the KIB application were predicted at the first-principles density functional theory (DFT) level.^23^ The bulk species such as K, Co, CoP, Co_2_P, and K_3_P were fully optimized under periodic boundary conditions until the energy converged to 10^−5^ eV and the force converged to 0.01 eV Å^−1^. The calculations were performed with the Perdew–Burke–Ernzerhof (PBE) exchange–correlation functional under the framework of the projected augmented wave (PAW) method^24,25^ implemented in the Vienna *ab initio* simulation package (VASP).^26^ The electronic wavefunctions were expanded using planewaves with an energy cutoff of 400 eV. For each calculation, the primitive cell was used as the initial geometry and a Monkhorst–Pack grid of *n*_*a*_ × *n*_*b*_ × *n*_*c*_, where *n*_*i*_ > 40 Å/*d*_*i*_ (*i* = *a*, *b*, and *c*, being the three axial directions, and *d*_*i*_ is the lattice parameter along the direction *i*),^27^ was used to sample the Brillouin zone. The slabs were built based on the optimized bulk structures. Slabs with certain surface terminations were cut from the bulk structure with a minimum thickness of 10 Å, expanded into supercells with lattice parameters *a* and *b* being no less than 10 Å, and extended by a vacuum of 15 Å along the *c* direction for the separation of the slab images. The constructed slabs were optimized with a constant supercell volume (*i.e.* with lattice parameters *a* and *b* relaxed).

To explore K^+^ extraction reaction, K^+^ ions near the surface of the optimized slabs were removed, and the resultant slabs were again optimized. To explore the K^+^ insertion on the CoP surfaces, K^+^ ions were added onto the surface one by one. Each additional K^+^ ion was placed at the most thermodynamically favorable site. To determine the site favorability for K^+^ insertion, a set of trial calculations were carried out. The slab primitive cell of the clean CoP surface was divided evenly by a 5 × 5 grid, and a K was placed at a grid point that was ∼2.0 Å above the slab surface to generate a trial configuration (out of 25 configurations).

To correlate the DFT results with the thermodynamic quantities, such as reduction potentials and CV spectra, from electrochemical measurements, the computational K^+^/K electrode model was adopted. In the computational K^+^/K electrode model, the reference state was set to the equilibrium state with Δ*G* = 0 for K(s) → K^+^ + e^−^. The reduction potential with respect to K^+^/K for the electrochemical reaction involving K^+^ + e^−^ could then be evaluated by substituting free energy of bulk K for free energy of (K^+^ + e^−^). Because no H atoms are involved in most of the reactions, for the sake of simplicity, the calculated electronic energies were used to approximate the Gibbs free energies.

## 3. Results and discussion

**Fig. 1:**
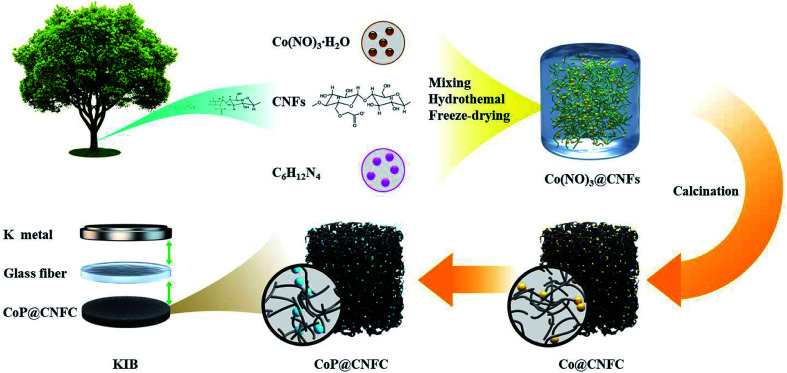
Schematic of the synthesis process of the robust CoP@CNFC composite *via* chemical bonding through a facile hydrothermal and subsequent *in situ* phosphidation approach.

The preparation procedure of CoP@CNFC is schematically depicted in Fig. 1. Briefly, a facile hybridization process was performed between cobalt nitrate and CNFs through the hydrothermal route, leading to electrostatic interaction among the positively charged cobalt ions and the negatively charged carboxyl and hydroxyl groups. As a result, cobalt ions were uniformly absorbed on the surface of the CNFs and a hybridized sol–gel of Co(NO_3_)_2_@CNFs was obtained. Then, after freeze-drying and annealing the sol–gel, the CNFs were converted to 3D network-like carbon (CNFC) and used to reduce the decomposed cobalt nitrate at high temperature, forming Co@CNFC with Co NPs immobilized in the carbon skeletons. By treating the Co@CNFC with an *in situ* phosphidation process, CoP@CNFC was finally synthesized. Owing to the unique interface joined *via* strong chemical bonding (electrostatic interactions and phosphatization reactions) between CoP and carbon, the structural stability of CoP@CNFC was largely enhanced, contributing to the efficient K-storage.

**Fig. 2:**
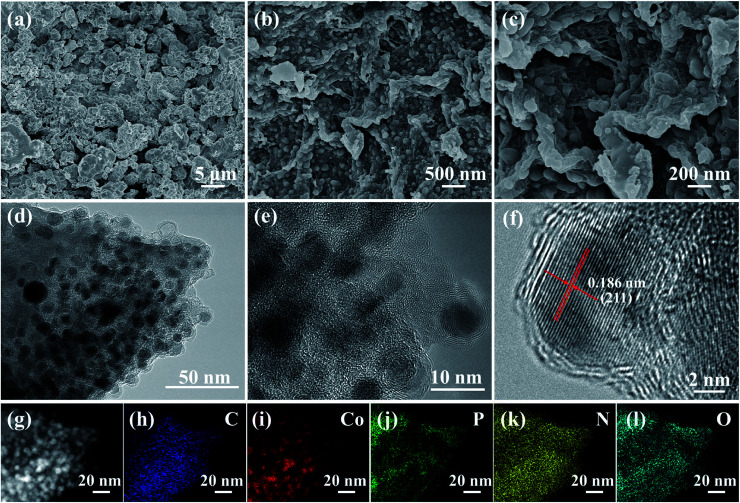
Morphology and microstructures of the CoP@CNFC composite: (a and b) SEM images, (c–e) TEM images, (f) HR-TEM image, (g–l) TEM-elemental mapping of C, Co, P, N, and O.

The morphologies and microstructures of the synthesized CoP and CoP@CNFC are shown in Fig. 2. The pure CoP prepared by direct phosphidation of Co under an identical annealing procedure exhibits large and severely agglomerated particles (Fig. 2a). In contrast, CoP NPs, which possess an average diameter of 5–25 nm, are uniformly immobilized in the CNFs-derived carbon sheets (CNFC) (Fig. 2b). Further observation (Fig. 2c) indicates that the skeletons of each CNFC are fully covered with CoP NPs on both sides, yielding a twisted and intertwined 3D network structure. The unique structure of CoP@CNFC is probably associated with the self-assembly of cross-linked CNFs under the effects of interfacial tension and hydrogen bonding during the freeze-drying process.^21^ The transmission electron microscopy (TEM) images of CoP@CNFC further reveal that the CoP NPs are well immobilized in the twisted and intertwined 3D carbon network (Fig. 2d, e and S1†). The high-resolution TEM image provides closer insight into the immobilized CoP NPs, which possess a lattice spacing of 0.186 nm, corresponding to the crystal plane of (211) (Fig. 2f). TEM-elemental mapping of CoP@CNFC was conducted using a random selected region; the results demonstrate that all of the contained elements of C, Co, P, N, and O have relatively uniform distributions in the composite (Fig. 2g–l).

**Fig. 3:**
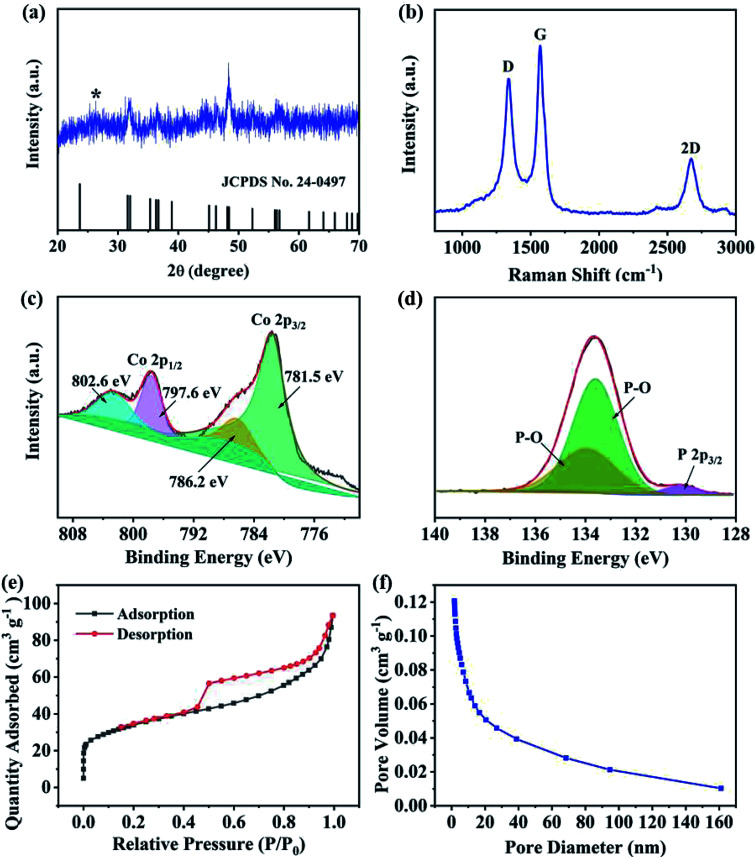
Phases, chemical states and porous profiles of the CoP@CNFC composite: (a) XRD patterns. (b) Raman spectrum. (c) High-resolution Co 2p XPS spectrum. (d) High-resolution P 1s XPS spectrum. (e) N_2_ adsorption–desorption isotherms. (f) Pore size distribution curve.

The crystal structure of CoP@CNFC was detected by X-ray diffraction (XRD) patterns (Fig. 3a). Most of the diffraction peaks are in good agreement with orthorhombic CoP (JCPDS no. 24-0497) except for a peak located at around 26.4°, which corresponds to the CNFC species.^28,29^ The associated crystalline structure of CoP in the composite possesses the lattice parameters of *a* = 5.077 Å, *b* = 3.281 Å, *c* = 5.587 Å and *α* = *β* = *γ* = 90°. The Raman spectrum of CoP@CNFC in Fig. 3b demonstrates three distinct peaks located at about 1338, 1570, and 2673 cm^−1^, referring to the D band, G band and 2D band, respectively.^30,31^ The intensity ratio of the D band to the G band (*I*_D_/*I*_G_) is calculated to be 0.8, which indicates a high graphitization extent of the structure, contributing to the achievement of high electrical conductivity for fast charge transfer in the electrode material.^32^

X-ray photoelectron spectroscopy (XPS) was conducted to investigate the elemental composition and surface chemical states of CoP@CNFC (Fig. S2†). The survey spectrum further reveals the existence of C, Co, P, N, and O elements (Fig. S2a†). Clearly, the C and O species originate from the carbonized CNFC, Co and N are introduced by the precursor of Co(NO_3_)_3_, and P originates from the phosphorous source of NaH_2_PO_2_·H_2_O. The obtained atomic ratios of C, Co, P, N and O are about 43.14%, 6.23%, 10.76%, 1.40% and 38.4%, respectively, which correspond to a mass ratio of 37.8 wt% of CoP in the composite (Table S1†). The high-resolution C 1s spectrum, shown in Fig. S2b,† can be differentiated into four peaks at 284.6, 285.1, 286.0 and 288.7 eV, belonging to C=C, C–C and C=N, C–O, and O–C=O, respectively.^33^ The high-resolution Co 2p spectra display the characteristic peaks of Co 2p_3/2_ and Co 2p_1/2_ (Fig. 3c), in which the peaks at 781.5 and 796.2 eV are assigned to the former and the peaks at 797.6 and 802.6 eV correspond to the latter.^34^Fig. 3d exhibits the high-resolution P 1s spectra consisting of three peaks, which are assigned to P 2p_3/2_ (130.2 eV) and oxidized P species (P–O) that resulted from oxidation of the surface in the air atmosphere (133.6 and 133.9 eV), corresponding to the generation of CoP.^30,35^ The N species were also investigated by XPS, as shown in Fig. S2c.† The three peaks at 399.5, 400.8 and 401.5 eV were fitted to pyridinic-N, pyrrolic-N and graphitic-N, respectively.^36,37^ It should be mentioned that the doping of nitrogen in the composite is beneficial to promote the electrical conductivity and increase the defects of the electrode, enabling the ionic reaction kinetics and more active sites for high K-storage capacity.^38^

N_2_ adsorption/desorption isotherms were also collected to examine the porous nature of CoP@CNFC. As shown in Fig. 3e, a combined type I/IV profile was demonstrated, indicating the existence of meso- and micro-porous structures.^39^ According to the Brunauer–Emmett–Teller (BET) method, the surface area of the composite was calculated to be 120.4 m^2^ g^−1^. The pore size distribution is displayed in Fig. 3f, which further verifies the centered porous nature of the meso- and micro-pores. The total pore volume was determined by the Barrett–Joyner–Halenda (BJH) method, and a considerable value of 0.12 cm^3^ g^−1^ was obtained. The unique porous feature of CoP@CNFC can certainly offer numerous reaction active sites and provide channels for fast K^+^ transport.^39^

**Fig. 4:**
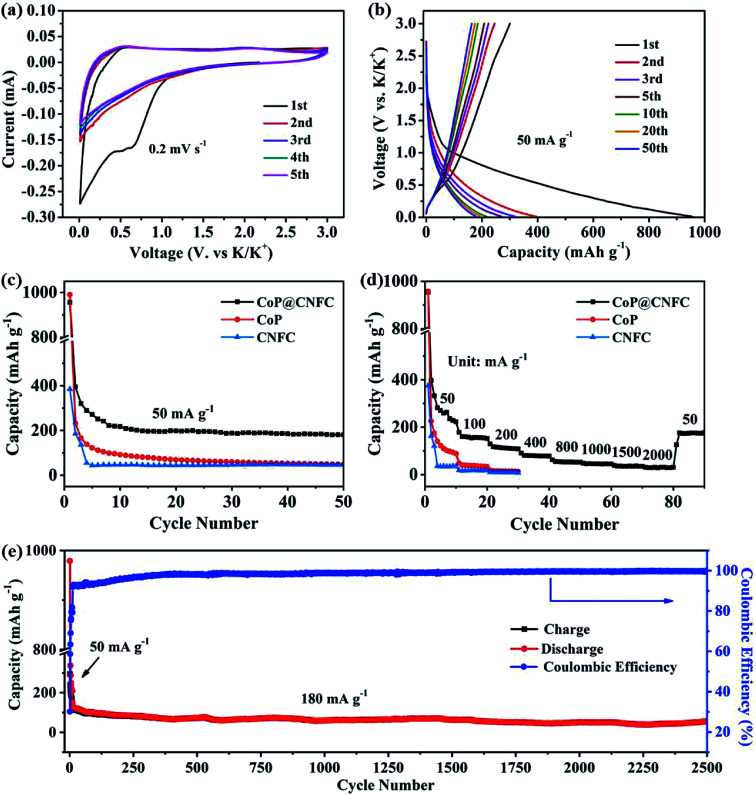
Electrochemical performance of the CoP@CNFC electrode in the voltage range of 0.01 and 3.0 V: (a) CV curves at a scan rate of 0.2 mV s^−1^. (b) Galvanostatic charge/discharge profiles for the 1^st^, 2^nd^, 3^rd^, 5^th^, 10^th^, 20^th^ and 50^th^ cycles at 50 mA g^−1^. (c) Cycling performance at 50 mA g^−1^. (d) Rate performance at various current densities ranging from 50 to 2000 mA g^−1^. (e) Long-term cycling performance at a higher current density of 180 mA g^−1^.

To investigate the K-storage behavior of CoP@CNFC, CR 2032 coin cells were assembled by employing K metal as the counter electrode. Fig. 4a displays the cyclic voltammetry (CV) profiles of the CoP@CNFC electrode for the initial five cycles in the voltage range of 0.01–3.0 V at a scan rate of 0.2 mV s^−1^. In the first cathodic scan, two reduction peaks at about 0.51 and 0.63 V are observed, which correspond to the inevitable formation of the SEI film and the partial conversion reaction of CoP on the electrode surface.^11,13,40,41^ In the first anodic scan process, two broad oxidation peaks that appeared at about 0.54 and 2.21 V can be ascribed to the extraction of K^+^ and the related transformation of K_*x*_P to CoP.^11,13,34,40–43^ In the following scans, the CV curves reveal almost identical reduction and oxidation peaks, suggesting high reversibility of the electrode during the potassiation/depotassiation process.^11,13,34,40–43^Fig. 4b exhibits the charge/discharge profiles of the electrode for the 1st, 2nd, 3rd, 5th, 10th, 20th and 50th cycles at a current density of 50 mA g^−1^. The electrode presents large charge/discharge capacities of 300.5/956.0 mA h g^−1^ at the first cycle, corresponding to a considerable initial coulombic efficiency (CE) of about 31.4%. The relatively large irreversible capacity loss in the first cycle is mainly related to the generation of an SEI film on the surface of the electrode.^11,13,34,40–43^ In the following cycles, the charge/discharge curves tend to overlap with each other; this is in good agreement with the CV results, manifesting the excellent reversibility and cycling stability of the electrode.^11,13,34,40–43^

The cycling performance of the electrode was initially evaluated at 50 mA g^−1^. As shown in Fig. 4c, CoP@CNFC delivers a high reversible capacity of 180.2 mA h g^−1^ after 50 cycles, which is significantly larger than those of its CoP and CNFC counterparts, further demonstrating the good cycling stability.^11,13,34,40–43^ The rate performance of CoP@CNFC and CoP at various current densities ranging from 50 to 2000 mA g^−1^ is displayed in Fig. 4d. It can be obviously observed that CoP@CNFC possesses better rate capability than CoP or CNFC. The discharge capacities of CoP@CNFC for 10 cycles are 222.8, 152.5, 108.6, 78.1, 52.6, 44.9, 34.9 and 30.8 mA h g^−1^ at the current densities of 50, 100, 200, 400, 800, 1000, 1500 and 2000 mA g^−1^, respectively. Notably, when the current density returns to 50 mA g^−1^, a considerable discharge capacity of 177.1 mA h g^−1^ can be recovered. In contrast, CoP delivers lower rate capacities than CoP@CNFC at all of the current densities. Impressively, CoP@CNFC also exhibits excellent long cycling performance at a higher current density of 180 mA g^−1^. As depicted in Fig. 4e, the electrode demonstrates a large reversible capacity of 56.7 mA h g^−1^ after 2500 cycles with an activation process in the first 5 cycles at 50 mA g^−1^ (in order to enable the formation of a stable SEI film and enhanced cycling stability of the electrode). The excellent cycling performance of CoP@CNFC is associated with its unique structure, with highly dispersed CoP NPs immobilized in the natural cellulose nanofiber network-derived carbon matrix (CNFC), which can provide robust structural stability and effective accommodation for K^+^ insertion/extraction during the cycling process.

**Fig. 5:**
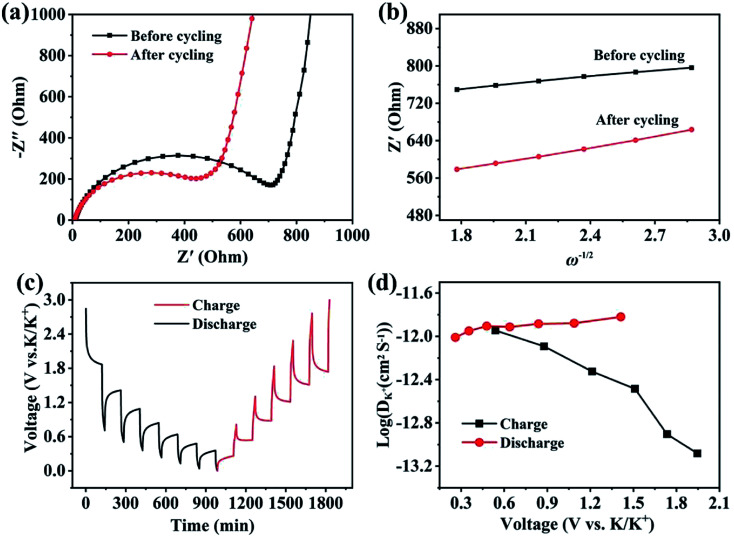
Electrochemical kinetics of the CoP@CNFC electrode: (a) EIS curve tested before cycling and after 200 cycles at 50 mA g^−1^. (b) Real parts of the impedance (*Z*′) *versus* the reciprocal square root of the lower angular frequency (*ω*) in the low frequency region. (c) Potential response during the GITT test at 50 mA g^−1^. (d) The logarithm of the diffusion coefficient for K^+^ ion (*D*_K^+^_) *versus* the voltage calculated based on the GITT profiles.

Electrochemical kinetic analysis was carried out to confirm the charge transfer and K^+^ diffusion in the CoP@CNFC electrode. As shown in Fig. 5a, the Nyquist plots of the electrode suggest identical features, including a straight sloping line in the low frequency region and a depressed semicircle in the middle–high frequency region, before cycling and after 200 cycles. Meanwhile, the charge transfer resistance (*R*_ct_) of the cycled electrode is obviously lower than that of the electrode without cycling, implying enhanced K-storage kinetics for the electrochemical reaction.^39,44^ Because the diffusion of K^+^ depends largely on the behavior in the low frequency region, the relationship of the real parts of the impedance (*Z*′) with the reciprocal square root of the angular frequency (*ω*) in the low frequency region was also determined. As shown in Fig. 5b, the cycled electrode displays a smaller slope than the electrode without cycling. Because the value of the Warburg factor (*σ*) is proportionate to the obtained slope and in inverse ratio to the square root of the diffusion coefficient of K^+^ (*D*_K^+^_),^39,45^ the cycled electrode is inferred to have a higher ionic diffusion coefficient. In other words, the diffusion of K^+^ in the electrode becomes easier upon cycling. In addition, the GITT curve was employed to measure the *D*_K^+^_ in the electrode with a pulse current at 50 mA g^−1^ for 20 min between rest intervals for 2 h (Fig. 5c). *D*_K^+^_ can be calculated based on Fick's second law according to the following simplified equation (eqn (1)).^46–48^1
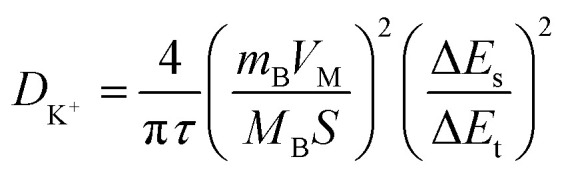
where *τ* refers to the constant current pulse time within one procedure, *m*_B_, *V*_M_, and *M*_B_ represent the weight, molar volume, and molar mass of the active material, respectively, *S* is the active surface area of the electrode, and Δ*E*_s_ and Δ*E*_t_ are the voltage change between the steady and original states at the plateau potential and the total voltage change during the current pulse *τ* excluding the *iR* drop, respectively. Fig. 5d reveals the *D*_K^+^_ obtained from the GITT curve at each discharge/charge state. It can be observed that the *D*_K^+^_ level of the electrode is relatively stable, with an order of about 10^−11^ cm^2^ s^−1^ in the discharge process. The highly dispersed CoP NPs immobilized in the CNFs-derived carbon along with the porous structure provide high conductivity, reduced diffusion paths and rich channels, which facilitate the diffusivity of K^+^ in the electrode.

**Fig. 6:**
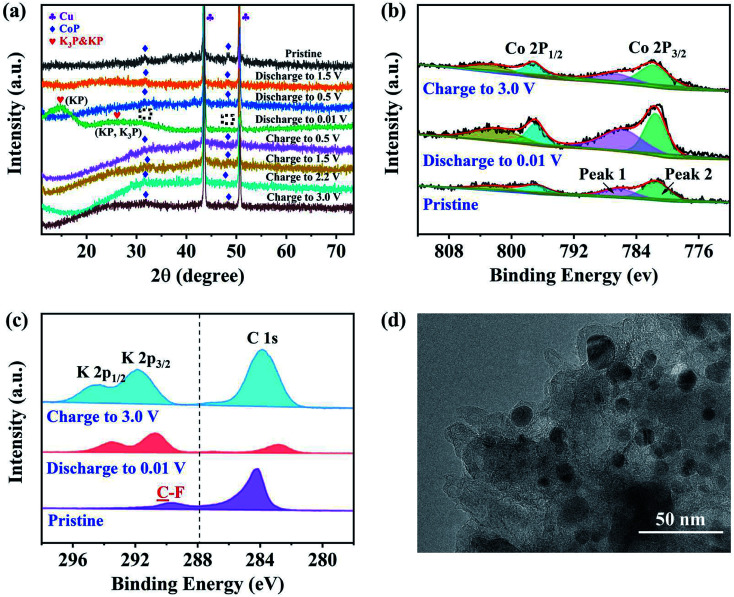
Phase and structure evaluation of the CoP@CNFC electrode: (a) *ex situ* XRD patterns of the electrode at different discharge and charge states. (b) *Ex situ* high-resolution XPS spectra of Co 2p collected in the pristine state after discharging to 0.01 V and after charging to 3.0 V, respectively. (c) TEM image of the electrode after 2500 cycles (charged state: 3.0 V). (d) TEM image of the cycled electrode (charged state: 3.0 V).

*Ex situ* XRD, XPS and TEM characterizations were performed to identify the phases and evaluate the structure of the CoP@CNFC electrode. Fig. 6a shows the *ex situ* XRD patterns of the electrode in different discharge and charge states. At the initial stage without discharging, the pristine electrode exhibits well-defined CoP peaks. Upon gradual discharge to 0.01 V, the CoP peaks decrease, indicating the occurrence of the electrochemical conversion reaction.^41,44,49^ At this stage, CoP is reduced (the products could be Co, Co_2_P, *etc.*). Uniquely, the CoP peaks almost disappeared at 0.01 V, and new distinct and wide peaks of KP (∼15°, 25°) and K_3_P (∼25°) were individually detected (the enlarged detail is shown in Fig. S3†). KP and K_3_P can be formed in the conversion of CoP to Co and Co_2_P upon potassiation, which implies a compositional change in the electrode material. Note that the disappearance of the CoP peaks does not necessarily imply full conversion of CoP due to a low signal-to-noise ratio of the *ex situ* spectra. In contrast, upon gradual charging from 0.01 to 3.0 V, the CoP peaks appear again and become increasingly more obvious, which is likely due to depotassiation. The increase of the CoP peaks is accompanied by the full disappearance of the KP and K_3_P-related peaks when the system is charged to a potential over 0.01 V (at 0.5, 1.5, 2.2 and 3.0 V), indicating that the compositional change of the anode material at 0.01 V is highly reversible.

The compositional evolution of CoP@CNFC was also characterized using *ex situ* XPS. Fig. 6b presents the high-resolution XPS spectra of Co 2p collected at the pristine stage, after discharging to 0.01 V and after charging to 3.0 V, respectively. The deconvoluted peaks (two Co 2p_2/3_ peaks at 797 and 802 eV) are essentially constant for the spectra of the three stages, suggesting the high electrochemical stability of the electrode material. The area ratio between the deconvoluted low-binding-energy (at 797 eV) and high-binding-energy (at 802 eV) Co 2p_3/2_ peaks was found to vary depending on the charge/discharge state. This low-to-high peak area ratio is 1.6 : 1 in the pristine CoP@CNFC. When discharged to 0.01 V, the area ratio increases to 1.7 : 1, implying a small decrease in the average Co 2p_3/2_ binding energy. A small portion of Co in the electrode may have been reduced. When charged to 3.0 V, conversely, the peak area ratio is 1.3 : 1, much lower than that for the pristine electrode, indicating a significant increase in the average Co 2p_3/2_ binding energy. This suggests that CoP is oxidized at 3.0 V. The K^+^ insertion/extraction of CoP@CNFC was monitored using *ex situ* high-resolution XPS spectra of K 2p. Fig. 6c shows that no K species were detected in the pristine electrode (only a C 1s peak in the adjacent binding energy range was found). During discharging, as K^+^ ions were gradually inserted into the electrode, the K 2p peak appeared and the peak intensified; eventually, a large ratio of K to C (high intensity of K in the XPS spectra) was observed at the fully discharged state (0.01 V). During charging, as K^+^ ions were extracted from the electrode, the K to C ratio decreased; at the fully charged state (3.0 V), a low ratio of K to C (low intensity of K in the XPS spectra) was obtained. It should be noted that a small amount of K species remained in the charged electrode due to slight irreversibility of the reaction,^44^ which is likely related to the formation of the SEI film. The above *ex situ* XRD and XPS observations suggest an essentially reversible conversion of CoP with K^+^ during the discharge and charge processes.^41,43^ Additionally, a TEM image of the cycled electrode (charged state: 3.0 V) is demonstrated in Fig. 6d; the active material of the electrode (CoP@CNFC) still maintains the designed structure after 2500 cycles, with CoP NPs well immobilized in the CNFs-derived carbon matrix (CNFC), which again demonstrates its rigid integrity for long-life cycling.

Additionally, to further understand the electrochemical insertion/extraction on the surface/interface of the CoP anode material during the discharge and charge states, first-principles calculations were carried out using the TH-2 JK supercomputer. By adopting the computational K^+^/K electrode model, the analysis and results with the thermodynamic quantities, such as reduction potentials and CV spectra from electrochemical measurements, were well correlated. The specific process is as follows. Firstly, the bulk-phase conversion reactions of CoP were considered (eqn (2)–(8)):

2 CoP(s) + 3K^+^ + 3e^−^ → K_3_P(s) + Co(s), *ε* = −0.10 V

3 2CoP(s) + 3K^+^ + 3e^−^ → Co_2_P(s) + K_3_P(s), *ε* = 0.04 V

4 CoP(s) + K^+^ + e^−^ = Co(s) + KP(s), *ε* = −0.33 V

5 2CoP(s) + K^+^ + e^−^ = Co_2_P(s) + KP(s), *ε* = 0.08 V

6 3CoP(s) + 2K^+^ + 2e^−^ = 3Co(s) + K_2_P_3_(s), *ε* = −1.34 V

7 6CoP(s) + 2K^+^ + 2e^−^ = 3Co_2_P(s) + K_2_P_3_(s), *ε* = −0.13 V

8 KP + 2K^+^ + 2e^−^ = K_3_P(s), *ε* = 0.02 V

Bulk species, including Co, K_3_P, Co_2_P, KP, and K_2_P_3_, can potentially be formed due to the charge/discharge of the electrode (Fig. S4†). The reduction potential *ε vs.* K^+^/K was obtained as the negative of the reaction energy divided by the number of electrons on the left-hand side of the reaction equation (−Δ*E*/*n* values are included in eqn (2)–(8)). Considering that experimentally, the battery was discharged to 0.01 V *vs.* K^+^/K, reactions with highly negative *ε* are not likely to occur. Therefore, bulk phase conversion reactions (3), (5), and (8) are most likely to occur at a potential close to the K^+^/K potential, forming Co_2_P, KP and K_3_P. No large-scale bulk conversion reactions are projected, as none of the surveyed reactions have a sufficiently positive potential. This also implies that the resultant bulk species at potentials near 0 V *vs.* K^+^/K may lack crystallinity. However, on a large time scale, such as the lifetime of a battery, extensive bulk-phase conversion with or without crystallization is still possible.

The energetics for the depotassiation of KP and K_3_P were investigated next. The reaction energies for the extraction of K^+^ from three different sites at or near the K_3_P (001) and KP (001) surfaces were predicted (Fig. S5†). The depotassiation potentials were evaluated to be −0.77, −1.02, and 0.56 V *vs.* K^+^/K for the three surface sites of K_3_P (001). Thus, most of the surface K^+^ of K_3_P can be facilely extracted under the operating conditions. The depotassiation potentials were evaluated to be 0.89, 0.96, and 1.11 V *vs.* K^+^/K for the three surface sites of KP (001) *vs.* K^+^/K. This indicates that the extraction of K^+^ from KP is more difficult than that from K_3_P, resulting in a lower energy density; however, it is still viable.

**Fig. 7:**
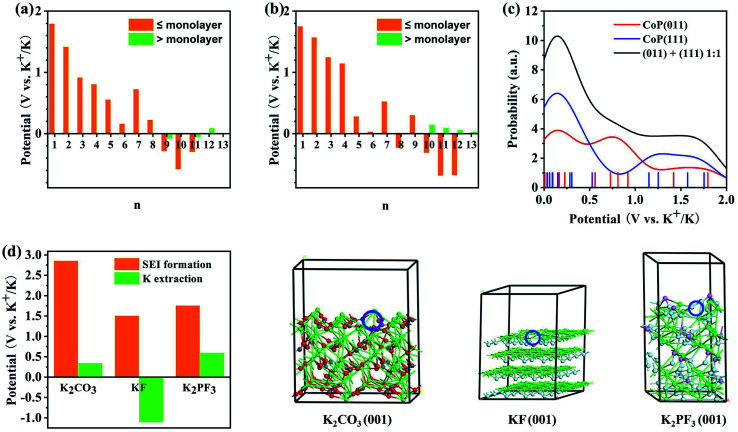
Theoretical analysis of the electrochemical reaction mechanism of the CoP@CNFC electrode based on first-principles calculations: (a and b) predicted reduction potentials for the insertion of *n*-th K onto (a) CoP (011) and (b) CoP(111) surfaces at the PBE level. The orange color indicates that the insertion leads to coverage less than or equal to a monolayer, and the green color indicates that the insertion leads to coverage greater than a monolayer. (c) Reduction probability convoluted with Gaussian functions with FWHM of 0.5 V based on the predicted reduction potentials for K^+^ insertion over the CoP (011) and (111) surfaces. (d) Comparison of the reduction potentials for the SEI film formation and K^+^ extraction for potential major SEI species. Atomic color code: green = K, gray = C, red = O, cyan = F, and pink = P. The K vacancy resulting from K^+^ extraction is indicated by the blue circle.

Potassiation during the charging process could also take place at the CoP electrode material. The K^+^ insertion reactions were investigated for the (011) and (111) surfaces of CoP using first-principles calculations. The reduction potentials for the insertion of the first K^+^ at various sites of the CoP (011) surface were found to be in the range of 1.18 to 1.80 V. Here, a higher potential indicates a more facile insertion. Additional K atoms were added to the most favorable configuration (Fig. S6†), and the predicted reduction potentials are shown in Fig. 7a. It was found that on a surface area of 148.9 Å^2^ (the supercell surface area for CoP (011)), a maximum number of 11 K atoms can be fitted into a monolayer; however, with a charging potential up to 0.01 V *vs.* K^+^/K, a 8-K monolayer is expected to be formed. When additional K^+^ was added as part of the second layer on top of the 8-K monolayer, the reduction potential were close to zero *vs.* K^+^/K and mostly negative, implying that a second layer of K atoms on CoP (011) may not be formed under the charging conditions. The insertion of the first K onto the CoP (111) surface exhibited reduction potentials in the range of 1.568 to 1.754 V at various surface sites. By incrementally adding K^+^ ions to the most favorable configuration (Fig. S7†), the reduction potential for each K^+^ insertion step on CoP (111) was evaluated, as shown in Fig. 7b. On a surface area of 148.9 Å^2^ (the supercell surface area for CoP (111)), a maximum number of 12 K atoms could theoretically be fitted into a monolayer, and under the charging conditions, a 9-K monolayer is expected on the basis of the predicted positive reduction potentials *vs.* K^+^/K. Further adding K atoms on top of the 9-K monolayer led to small positive reduction potentials *vs.* K^+^/K; thus, K^+^ could be further accumulated over CoP (111) under the experimental charging conditions, which implies that the CoP (111) surface may contribute more to the energy density of the battery than the CoP (011) surface, which may be due to the different lattice spacings of the two surfaces. The insertion and extraction of K^+^ over the CoP surfaces are likely reversible, and the predicted reduction potential range matches the distribution of CV peaks during the cathodic scan. Fig. 7c shows the probability distribution of the K insertion reaction as a function of the reduction potential, convoluted using Gaussian functions with FWHM = 0.5 V (full with at half maximum) on the basis of the predicted reduction potentials. The shape of the probability distribution for combining the distribution functions of CoP (011) and (111) at a 1 : 1 ratio was found to be highly comparable to the CV spectrum shape during the cathodic scan. This suggests that the K^+^ insertion mechanism of the CoP in KIBs is dominated by the insertion of K over the CoP surfaces.

According to the experimental CV curves, an SEI film was formed during the first cathodic scan cycle. Possible reactions between the K^+^ ions and the electrolytes, including EC, DEC, and KPF_6_, were considered (Fig. S8†). The reaction of K^+^ with EC can lead to the formation of (CH_2_OCO_2_K)_2_ and C_2_H_4_,^50^ for which the reduction potential is predicted to be 1.45 V *vs.* K^+^/K (eqn (9)). K^+^ can also react with DEC to form K_2_CO_3_, utilizing a reduction potential of 2.86 V (eqn (10)). The highly positive potential implies that these conversions are irreversible. KPF_6_ can dissociate exothermically into solid-phase KF and PF_5_ (eqn (11)) with a reaction energy of −0.56 eV. This reaction, despite appearing to be spontaneous, may be kinetically difficult due to barriers for the ion dissociation and phase transition. K^+^ can react with PF_5_ to form K_*x*_PF_5−*x*_, *x* = 1–5, stepwise (eqn (12)–(16)):

9 2EC + 2K^+^ + 2e^−^ → (CH_2_OCO_2_K)_2_ + C_2_H_4_, *ε* = 1.45 V

10 2DEC + 2K^+^ + 2e^−^ → K_2_CO_3_(s) + C_4_H_10_, *ε* = 2.86 V

11 KPF_6_ → KF(s) + PF_5_(s), Δ*E* = −0.56 eV

12 PF_5_(s) + 2K^+^ + 2e^−^ → KPF_4_(s) + KF(s), *ε* = 1.51 V

13 KPF_4_(s) + 2K^+^ + 2e^−^ → K_2_PF_3_(s) + KF(s), *ε* = 1.76 V

14 K_2_PF_3_(s) + 2K^+^ + 2e^−^ → K_3_PF_2_(s) + KF(s), *ε* = −0.80 V

15 K_3_PF_2_(s) + 2K^+^ + 2e^−^ → K_4_PF(s) + KF(s), *ε* = 3.11 V

16 K_4_PF(s) + 2K^+^ + 2e^−^ → K_5_P(s) + KF(s), *ε* = 0.20 V

It appears that the stepwise reaction route led to the formation of KF, KPF_4_ and K_2_PF_3_, while further potassiation was blocked by the negative reduction potential for converting K_2_PF_3_ into K_3_PF_2_. However, the formation of K_3_PF_2_, K_4_PF, or K_5_P cannot be ruled out under the experimental conditions, as the reduction potentials for forming the K_*x*_PF_5−*x*_ species directly from KPF_6_ are all positive, as shown by eqn (17)–(21):

17 KPF_6_ + 2K^+^ + 2e^−^ → KPF_4_(s) + 2KF(s), *ε* = 1.80 V

18 KPF_6_ + 4K^+^ + 4e^−^ → K_2_PF_3_(s) + 3KF(s), *ε* = 1.78 V

19 KPF_6_ + 6K^+^ + 6e^−^ → K_3_PF_2_(s) + 4KF(s), *ε* = 0.92 V

20 KPF_6_ + 8K^+^ + 8e^−^ → K_4_PF(s) + 5KF(s), *ε* = 1.47 V

21 KPF_6_ + 10K^+^ + 10e^−^ → K_5_P(s) + 6KF(s), *ε* = 1.21 V

Note that the reduction potentials for the direct reactions of K^+^ with KPF_6_ may not be significantly meaningful, as these reactions include kinetically difficult phase-transition steps.

The reduction potentials for the K^+^ extraction reactions of the major possible constituents of the SEI films (K_2_CO_3_, KF, and K_2_PF_3_) were also predicted, as shown in Fig. 7d. The results show that the reduction potentials for surface K^+^ extraction of these species are much lower than the corresponding SEI formation potentials. This corroborates the irreversible electrochemical behaviors of the SEI species. Overall, the CV peaks that appeared during the first cathodic scan cycle and disappeared in the succeeding cycles (at 0.51 and 0.63 V) can be attributed to the irreversible reactions of K^+^ with EC, DEC, and KPF_6_ to form the SEI films, which were likely composed of (CH_2_OCO_2_K)_2_, K_2_CO_3_, KP, K_3_PF_2_, PF_5_, and K_5_P. There are differences of over 1.0 V between the predicted reduction potentials for SEI film formation and the experimentally observed values (Fig. 4a), which can be ascribed to the activation energies for the related diffusion and phase-transition processes.

Clearly, the above theoretical analysis and experimental results show a high degree of consistency, which can be combined to reveal the electrochemical reaction mechanism of the electrode material. The deep elucidation of the reversible processes of K^+^ insertion and extraction on the surface/interface of the CoP active material during the discharge and charge states clearly highlights the significance of the material for efficient K-storage.

## 4. Conclusion

In summary, we propose a novel CoP@CNFC composite, synthesized through a facile hydrothermal and subsequent *in situ* phosphidation process, as an anode material for stable storage of K-ions. Benefiting from the unique design of a rigid structure composed of highly dispersed CoP NPs immobilized in natural cellulose nanofiber network-derived carbon through strong interface joins *via* chemical bonding, the as-synthesized composite can provide diverse merits, including fast charge transportation, sufficient active sites, and effective accommodation for K^+^ insertion/extraction. As a result, the CoP@CNFC electrode delivers considerable specific capacity, enhanced rate capability and excellent cycling stability. Further *ex situ* characterizations and theoretical simulations of CV and SEI profiles based on first-principles calculations uncovered the electrochemical reaction mechanism of the electrode material and deeply elucidated the reversible process of K^+^ insertion and extraction on the surface/interface of the CoP active material, highlighting its significance for efficient K-storage. This work provides new insights into the exploration and understanding of advanced TMP-based anode materials for rechargeable KIBs.

## Conflicts of interest

There are no conflicts to declare.

## Supplementary Material

RA-010-D0RA09478A-s001
