# Supplementary material for: Achieving the robust immobilization of CoP nanoparticles in cellulose nanofiber network-derived carbon via chemical bonding for a stable potassium ion storage
Source: RSC Adv. 2020 Dec 17;10(72):44611–23. doi: 10.1039/d0ra09478a (PMC9058511; doi:10.1039/d0ra09478a)
Supplement: RA-010-D0RA09478A-s001 [file RA-010-D0RA09478A-s001.pdf]

Electronic Supplementary Material (ESI) for RSC Advances.
This journal is © The Royal Society of Chemistry 2020

Supporting Information

Achieving robust immobilization of CoP nanoparticles in cellulose nanofiber networks-derived carbon via chemical bonding for stable potassium-ion storage

Xudong Zhao[1‡], *Dan Zhou*[1‡*], *Mingyang Chen*[2,4,5*], *Jiaqi Yang*[3], *Li-Zhen Fan*[1*]

[1] Center for Green Innovation, School of Mathematics and Physics & Beijing Advanced Innovation Center for Materials Genome Engineering, University of Science and Technology Beijing, Beijing 100083, China

[2] Center for Green Innovation, School of Materials Science and Engineering, University of Science and Technology Beijing, Beijing 100083, China

[3] Office of Educational Administration, Shenyang Open University, Shenyang 110003, China

[4] Shunde Graduate School of University of Science and Technology Beijing, Foshan 528000, China

[5] Beijing Computational Science Research Center, Beijing 100084, China

‡These authors contributed equally to this work.

*Corresponding authors: zhoudan@ustb.edu.cn (D. Zhou); mychen@ustb.edu.cn (M. Chen); fanlizhen@ustb.edu.cn (L.-Z. Fan).

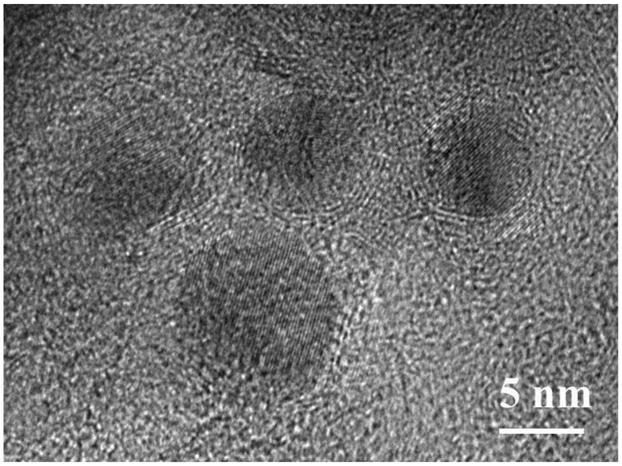

Fig. S1 TEM image of the CoP@CNFC composite.

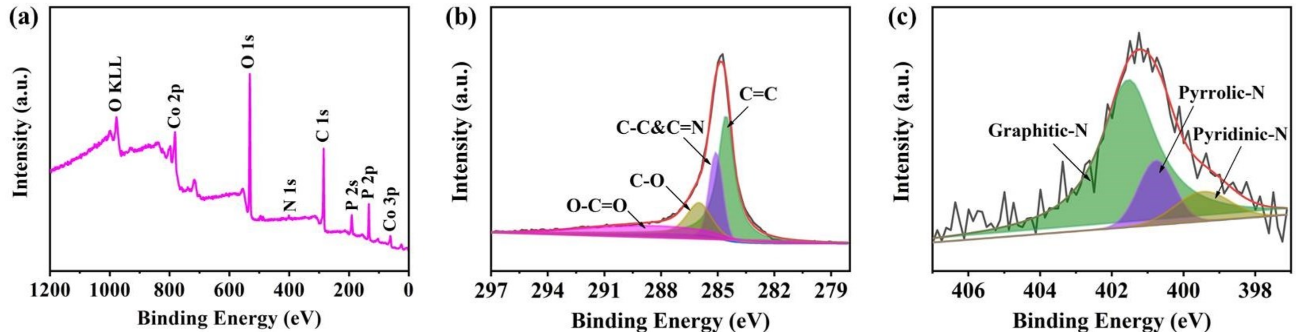

Fig. S2 (a) X-ray photoelectron spectroscopy (XPS) spectra of CoP@CNFC. (b) High-resolution C 1s. (c) High-resolution N 1s.

Table S1 Elemental content analysis result of the CoP@CNFC composite

Elements	Atomic Mass	Atomic Percentage (at%)	Mass content (wt%)
C	12.01	43.14	28.0
N	14.00	1.40	1.10
Co	58.93	6.23	19.8
P	15.98	10.76	18.0
O	16.00	38.46	33.1

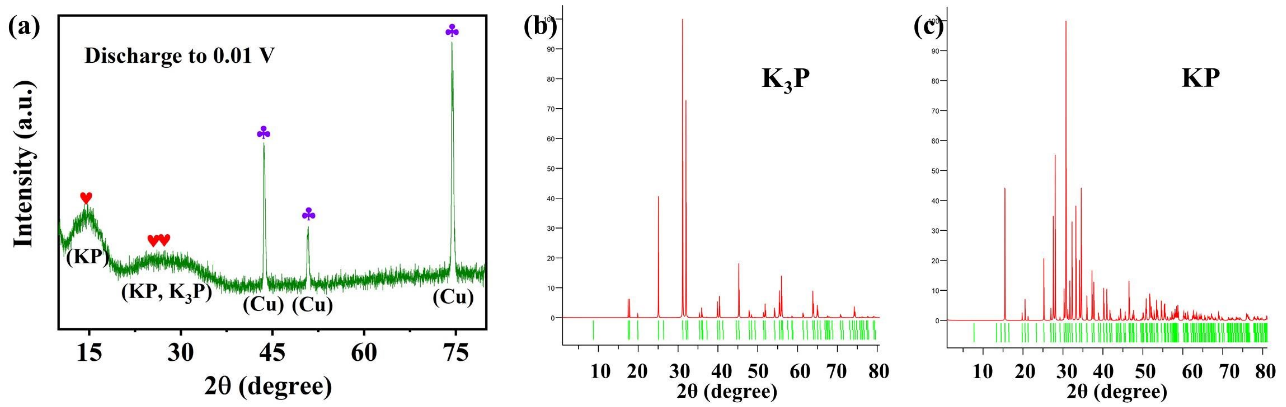

Fig. S3 (a) *Ex-situ* XRD patterns of the electrode discharged to 0.01 V. (b, c) the corresponding XRD patterns of the generated K_3P and KP at the fully discharged stage (0.01V).

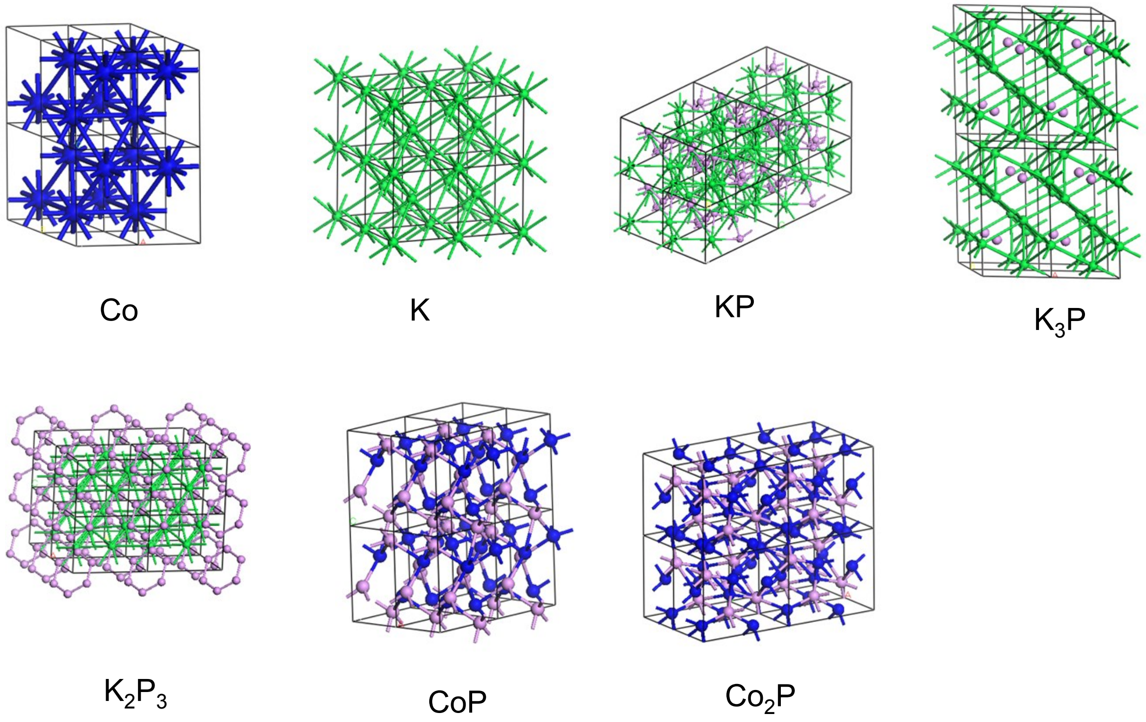

Fig. S4. Optimized bulk species related to the CoP-K-ion battery at the PBE level. Color code: green = K, pink = P, and blue = Co.

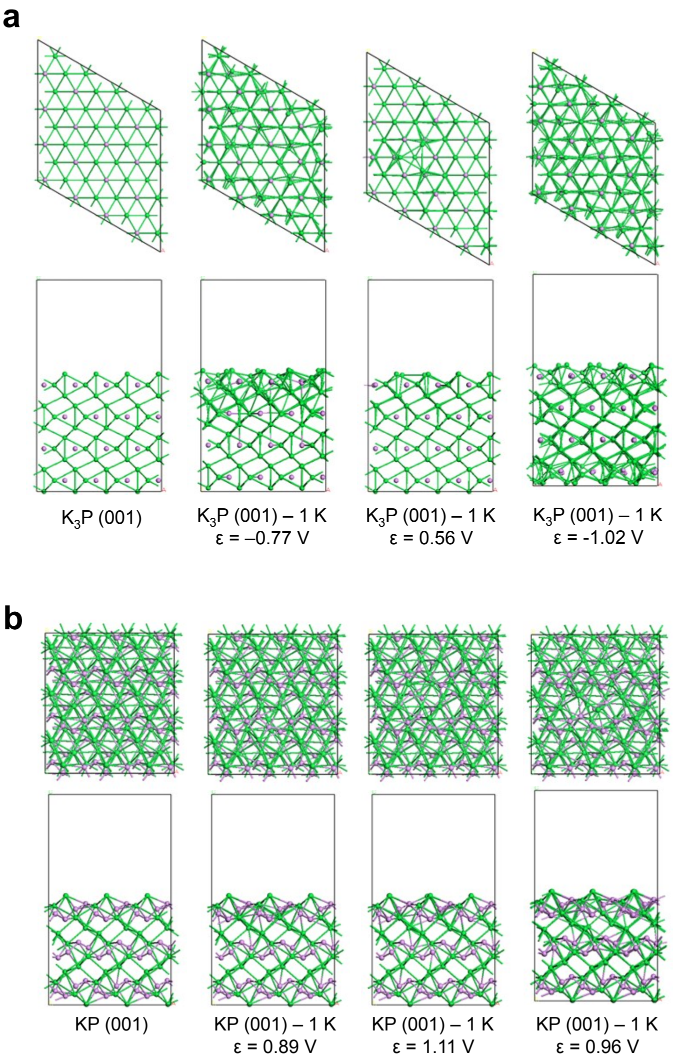

Fig. S5. Optimized geometries and predicted reduction potentials vs. K^+/K for (a) K_3P (001) and (b) KP (001) surfaces at the PBE level. Color code: green = K, and pink = P.

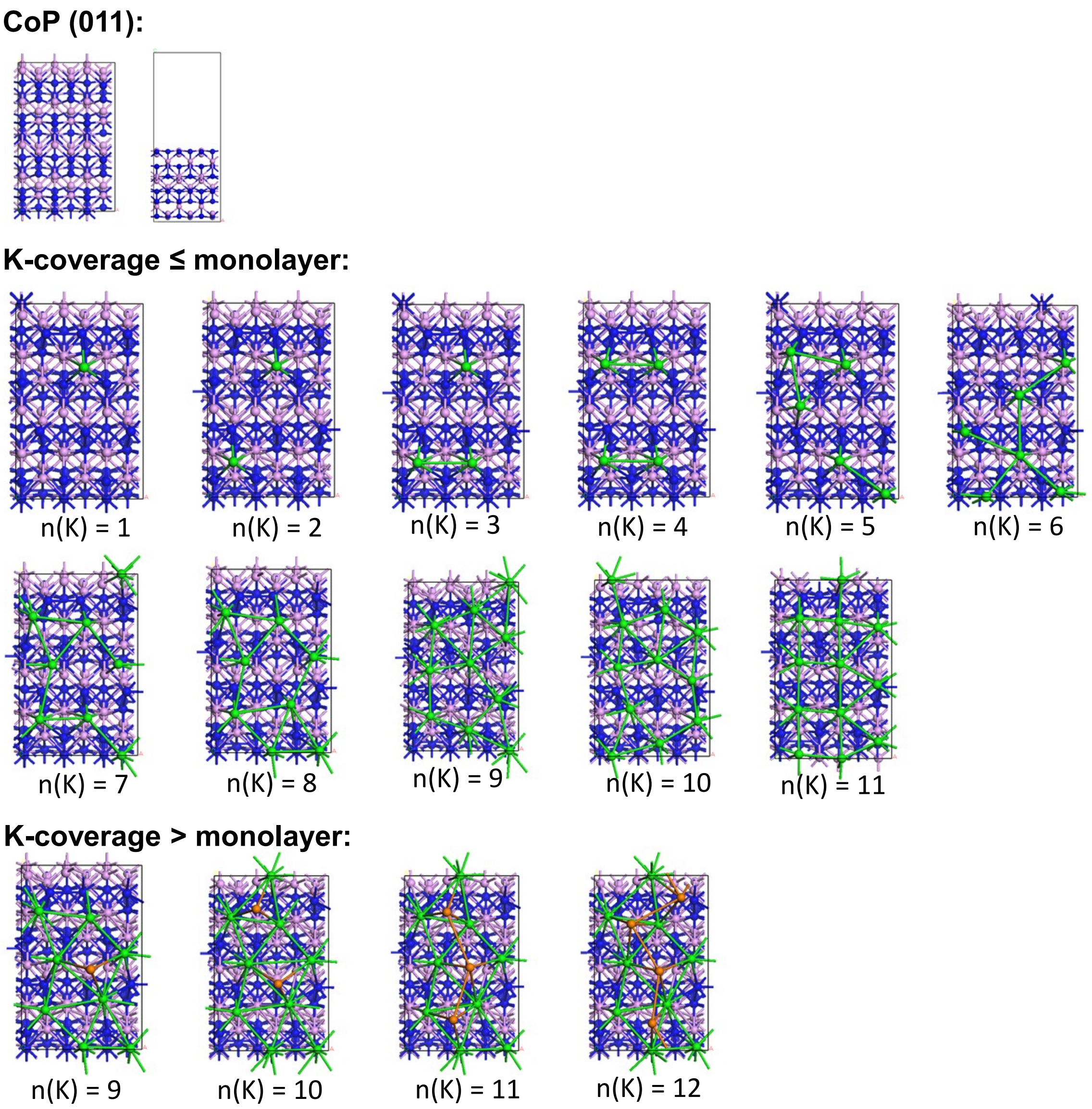

Fig. S6. Optimized geometries for potassiation of the CoP(011) slab. The surface area of the slab is 147.0 Å^2. Color code: green = K (first layer), orange = K (second layer), pink = P, and blue = Co.

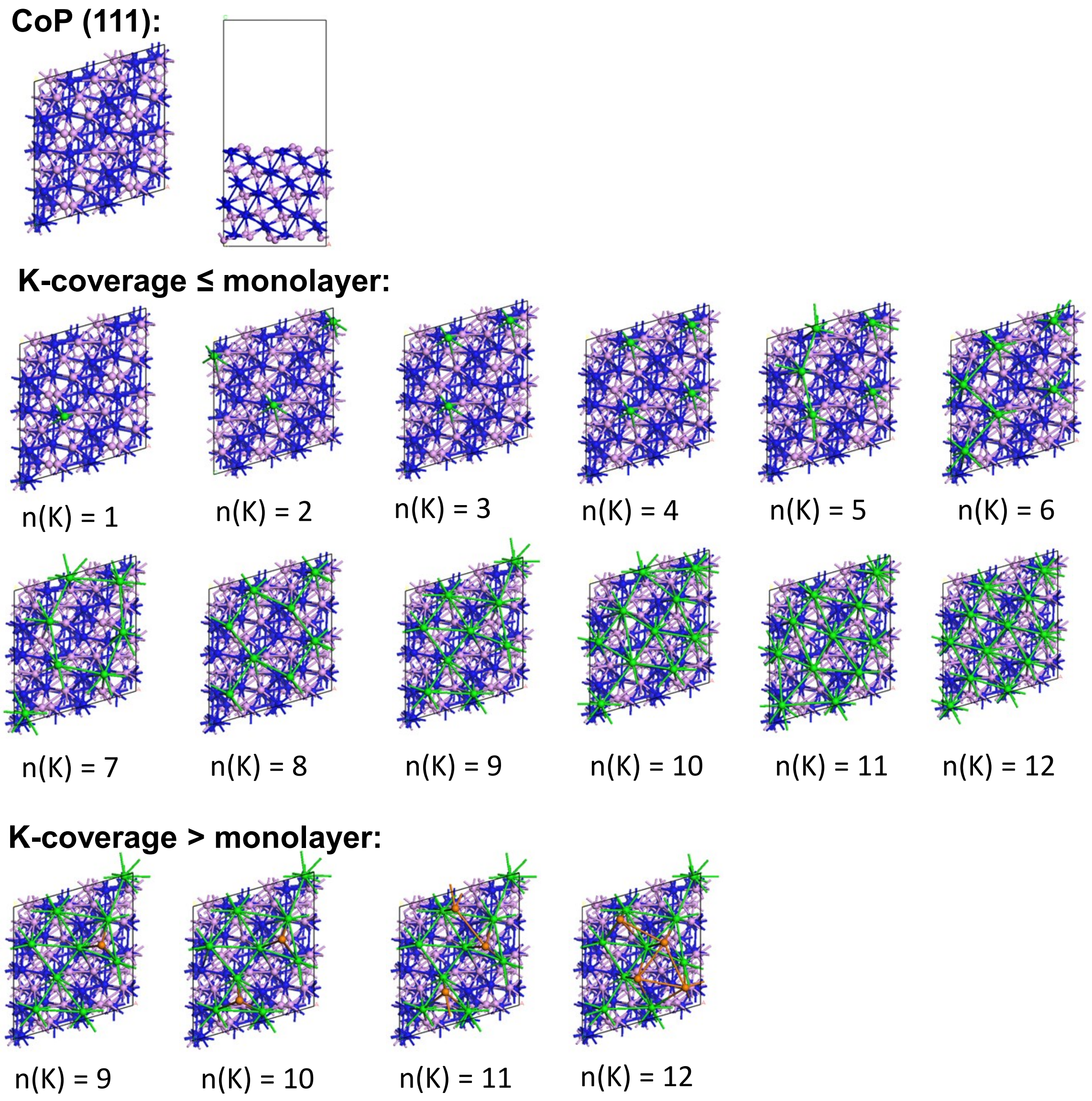

Fig. S7. Optimized geometries for potassiation of the CoP(111) slab. The surface area of the slab is 148.9 Å^2. Color code: green = K (first layer), orange = K (second layer), pink = P, and blue = Co.

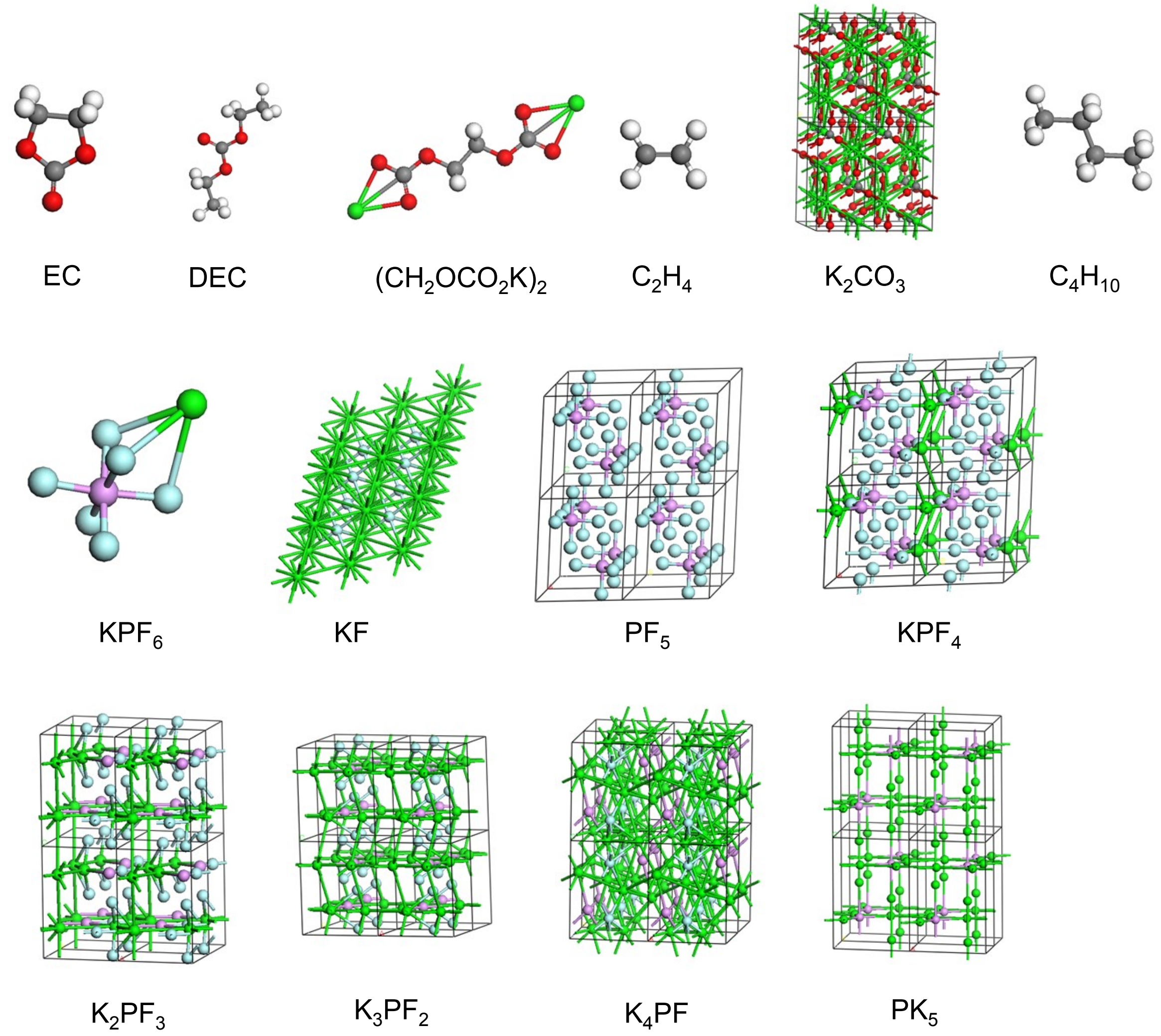

Fig. S8. SEI-related Species for K-ion battery with EC, DEC, and KPF_6 electrolytes.
